# An exploratory study to evaluate visual function endpoints in non-advanced age-related macular degeneration

**DOI:** 10.1186/s12886-020-01683-8

**Published:** 2020-10-22

**Authors:** Divya Narayanan, John Rodriguez, Garrick Wallstrom, Donna Welch, Matthew Chapin, Paul Arrigg, Mark Abelson

**Affiliations:** 1 Ora, Inc, 300 Brickstone Square, Andover, MA 01810 USA; 2 Statistics and Data Corporation, Tempe, AZ USA; 3 Joslin Diabetes Center, Boston, USA; 4 Ophthalmology, Harvard Medical School, Boston, MA USA; 5 Ophthalmology, Massachusetts Eye and Ear, Boston, MA USA

**Keywords:** Visual function, Endpoint, Contrast, Flicker, Reading, AMD

## Abstract

**Background:**

To prevent irreversible vision loss in age-related macular degeneration (AMD), it is critical to detect retinal dysfunction before permanent structural loss occurs. In the current study we evaluated a series of visual function tests to identify potential endpoints to detect visual dysfunction in non-advanced AMD.

**Methods:**

A series of visual function tests were performed on 23 non-advanced AMD subjects (AREDS grade 1–4 on simplified scale) and 34 age-matched normals (AREDS grade 0). Tests included some commonly used endpoints such as ETDRS visual acuity (VA), low luminance (LL) 2.0ND ETDRS VA, MNREAD as well as newly developed tests such as the Ora-VCF™ test, Ora-tablet reading test, color sensitivity etc. Differences between the two groups were compared for each test. Test-retest repeatability and reproducibility was assessed on a subset of subjects and percent agreement was calculated.

**Results:**

There was no difference in standard ETDRS VA between non-advanced AMD (0.06 ± 0.02 logMAR) and normal groups (0.04 ± 0.02 logMAR) (*p* = 0.57). LL 2.0 ETDRS VA and MNREAD showed no difference between the groups (*p* > 0.05). Ora-VCF™ test was significantly worse in the non-advanced AMD group compared to normals (0.67 ± 0.07 in AMD; 0.45 ± 0.04 in normals, *p* = 0.005). Non-advanced AMD subjects also had significantly worse reading performance using the Ora-tablet with LL 2.0ND (114.55 ± 11.22 wpm in AMD; 145.17 ± 9.55 wpm in normals *p* = 0.049). No significant difference between the groups was noted using other tests. Repeatability was 82% for Ora-VCF™ test and 92% for Ora-tablet LL 2.0ND reading. Reproducibility was 89% for both Ora-VCF™ test and Ora-tablet LL 2.0ND reading.

**Conclusion:**

While there was no significant difference between non-advanced AMD and normal groups using some current common endpoints such as ETDRS VA, LL 2.0 ETDRS VA or MNREAD, Ora-VCF™ test and Ora-tablet LL 2.0ND reading tests were able to identify significant visual dysfunction in non-advanced AMD subjects. These tests show promise as endpoints for AMD studies.

## Background

Age-related macular degeneration (AMD) is the leading cause of visual impairment in developed countries [1, 2]. With an increase in aging population, the prevalence rate of AMD is expected to rise steeply and predicted to reach 288 million globally by 2040 [3]. While the actual cause of the disease remains unclear, several risk factors have been identified. Age, ethnicity and genetics are all known to play important roles. The Beaver Dam Eye Study showed that the development of AMD over a 10 year period was 4.2% for persons aged 43 to 54 years and was 46% for those aged 75 years or older [4]. Observations from the Baltimore Eye Study suggests that late-stage AMDs are more common among Caucasians [5]. Genetic studies have identified several genetic factors linked to AMD. Several studies have found a strong association between complement factor H polymorphism (Y402H) and higher risk of AMD [6, 7]. Several modifiable risk factors such as smoking and diet have also been identified [8].

Early stage AMD is characterized by basal laminar and/or drusenoid deposits seen in the retinal pigment epithelium (RPE) or sub RPE layer with minimal or no impact on standard visual acuity (VA) [9]. More advanced forms of the disease, during which significant loss of VA occurs, involves loss of photoreceptors and RPE layers resulting in geographic atrophic (GA) in dry AMD or rapid growth of the neovascular membrane into the sensory retina in wet AMD. With the advent of anti-vascular endothelial growth factor (anti-VEGF) treatments more than a decade ago, significant improvements have been made in managing acute vision loss that occurs in wet AMD [10]. However, there are no treatments currently approved for dry AMD [11–13]. Two major phase 3 studies Chroma and Spectri that investigated therapeutic effects of Lampalizumab, a complement D monoclonal antibody, on reducing the growth of GA failed to meet its primary endpoint [14]. Similar phase 3 studies on evaluating other compounds to reduce GA growth are currently under trial (example APL-2) [15]. Currently the standard of care for dry AMD is a recommendation for antioxidant supplementation and periodic retinal exam to detect development of wet AMD. While preventing further progression during advanced stages of the disease is important, it is unclear if visual function could be preserved and maintained after such permanent structural damage.

Our working hypothesis is that treatments initiated during earlier stages of AMD have the best possibility to prevent permanent retinal structural damage from occurring. Given the extent of irreversible retinal damage that occurs in late stage dry AMD, it is likely that retinal neurons are experiencing dysfunction during the preceding stages (i.e. sick but not dead). With appropriate intervention during these earlier stages, this dysfunction could be possibly reversed in dry AMD. Treatment with a high dose of atorvastatin in non-advanced AMD subjects has shown to result in regression of drusen deposits [16]. Similarly, carotenoid supplementation in early AMD has been shown to improve the macular pigment optical density (MPOD) [17]. A major hurdle in the development of novel therapeutics for early AMD is the lack of sensitive endpoints to assess changes in visual function during the early stages of the disease [18, 19]. Visual acuity (VA) is the most commonly used metric to assess visual function. However, VA remains minimally affected in dry AMD during the earlier stages until GA occurs. In the AREDS 10 year follow-up study, among the subjects who did not progress to advanced AMD, the median VA worsened only by a few letters and remained 20/25 even after 10 years [20].

Given that the greatest risk factor for the development of AMD is aging, it is not surprising that a lot of pathological changes and functional deficits that occur in the early stages of AMD also occur in older normals. Our goal was to develop functional tests that are capable of identifying subtle differences between these two groups and reliably capture underlying neuronal dysfunction in AMD at its earliest stage. While several previous studies have looked into sensitive visual function tests for early and intermediate stages of AMD [18, 21–23], our study is unique in that we focused on identifying tests that are sensitive but also relatively easy and simple to implement in clinical trial settings by focusing on cone-based tests. The test duration for each of the visual function tests described in this study is less than 5 min per eye. While identifying a sensitive test is critical, it is also crucial to develop a test that is practical to administer in clinical trial setting and easy for the subject and technician to use. This ensures better subject and site compliance and thereby the clinical application and usefulness of the test in a therapeutic trial. In the current study we evaluated a series of newly developed functional tests on a cohort of non-advanced dry AMD subjects and age-matched normal controls. Additionally we also tested some current commonly used endpoints on the same cohort for comparison.

## Methods

The study protocol was approved by an independent Institutional Review Board (Alpha IRB, San Clemente, CA, USA) and all subjects provided written informed consent. The study was conducted in accordance with the ethical principles of the Declaration of Helsinki.

### Sample size

This was an exploratory study designed to evaluate visual function tests to identify potential endpoints to detect visual dysfunction in non-advanced AMD. As such, generic (i.e., non-test specific) sample size calculations were made, based on detecting a Cohen’s D effect size of 1.0 using a two-sided two-sample t-test and significance level α = 0.05. The number of subjects required for 80% power was 16 per group.

### Subjects

All subjects were required to be 60 years or older and be willing and able to perform all study procedures. Subjects were excluded from the study if they had a history of seizures or epilepsy, had a diagnosis or evidence of advanced AMD (GA or wet AMD), significant cataracts, history of ocular trauma or surgery (except cataract surgery) or other retinal diseases in the study eye. During the initial screening visit all subjects underwent informed consent, detailed medical and ocular history, visual acuity testing using ETDRS chart, potential acuity meter (PAM), optical coherence tomography (OCT) and dilated fundus photos. While this criterion was not used for subject inclusion or exclusion, based on medical histories documented during the screening visit, none of the subjects were on any form of carotenoid supplementation.

### Retinal imaging and fundus grading

During the initial visit, macular scans were obtained in both eyes using spectral domain OCT (Spectralis, Heidelberg, Germany). Both eyes were then dilated using 1% tropicamide and 2.5% phenylephrine. After optimal pupil dilation (> 7 mm diameter) digital color fundus photos were taken (450 plus camera; Carl Zeiss Meditec). Photos were evaluated by a retinal specialist ophthalmologist and graded using the AREDS simplified grading [24]. A grade of 0, 1, 2, 3 or 4 was assigned for each subject. In addition, the ophthalmologist also used multimodal imaging of color fundus photos, OCT images and infrared fundus images to specifically look for presence or absence of reticular pseudo drusen (RPD). The ophthalmologist concluded that based on the multi-modal images reviewed no RPD was detectable for any subjects included in the cohort.

### Grouping

**Fig. 1:**
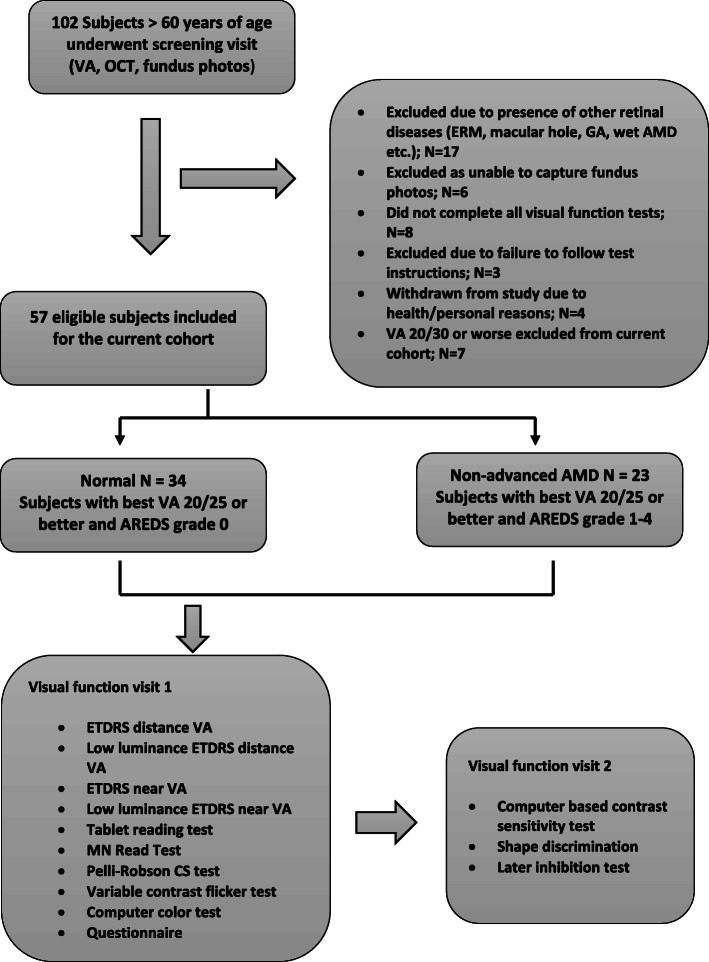
Flow chart describing clinical process

For each subject, based on best visual acuity (best of ETDRS VA and PAM) and fundus grading, a qualifying eye was designated as the study eye. If both eyes of a subject qualified then one eye was selected at random as the study eye by the examiner. A total of 102 subjects were originally screened for the study. After excluding subjects who screen failed or failed to complete all study procedures, 57 subjects completed all study procedures over 2 to 3 follow-up visits. The focus of the study was to compare two groups; 1) Normal control group defined as subjects with best VA 20/25 or better and AREDS grading of 0 (*N* = 34) and 2) Non-advanced AMD group defined as subjects with best VA 20/25 or better and fundus grading of AREDS ≥1 (*N* = 23). Among the 23 subjects in the non-advanced AMD group, 8 subjects had AREDS grade 1, 10 subjects had AREDS grade 2, one subject had AREDS grade 3 and four subjects had AREDS grade 4. Since the AREDS simplified grading uses a bilateral grading scheme, when subjects were classified using Beckman scale as described in Ferris 2013 [25], then 12 subjects had early AMD and 11 had intermediate AMD in the study eye in our cohort. Figure 1 outlines the study flow process.

### Visual function tests

A series of visual function tests were performed during follow-up visits. Both normal and AMD subjects were tested in a similar manner and sufficient breaks were given between each test to avoid fatigue and to allow for recovery from previous tests. All tests were performed monocularly for the study eye with appropriate correction. The fellow eye remained occluded throughout the testing.

Two examiners were responsible for administering all visual function for all subjects. About half of the tests were done by examiner 1 and the other half were done by examiner 2. Both the examiners were trained and signed off by the study investigator to be equally competent and reliable to administer the tests and followed the exact same written statement of procedures. For the repeatability and reproducibility portion of the study, only examiner 1 was involved in testing for both sessions on both days.

#### Standard ETDRS visual acuity and low-luminance (LL) 2.0 ND ETDRS visual acuity

ETDRS distance visual acuity as well as LL visual acuity (using 2.0 ND) was tested [26]. All subjects underwent standard ETDRS test first followed by the low luminance ETDRS VA. Subjects used an occluder to cover the non-tested eye and were asked to read letters from the ETDRS chart left to right and top to bottom. VA was calculated based on number of letters read correctly.

#### Ora-tablet Reading

Reading tests were performed using an electronic tablet. The tablet was placed on a tablet stand which was rested on a table and at eye level of the subject. The test subjects were seated at 40 cm distance from the table. All subjects were best corrected for 40 cm working distance for this test. The test was done monocularly and the non-tested eye remained occluded throughout the test. The test examiner was seated next to the subject. Prior to starting the test, all subjects were given the exact same instructions that they will be performing a series of reading tests under various challenging conditions; their reading speed and accuracy will be measured. A total of 7 different passages were presented in the following order; high contrast high luminance (HCHL), mesopic LL (2.0 ND and < 1% transmittance), low contrast, crowding, pepper words and vanishing optotypes. Time taken to read each passage accurately was calculated in words per minute (wpm).

#### MNREAD

The MNREAD test was performed under standard condition as well as under low-luminance (with 2.0 ND) using a MNREAD paper chart at 40 cm. Reading speed and accuracy for each font size was measured. Maximum reading speed in words per minute (wpm) and reading acuity were calculated.

#### Ora-VCF™ test

This was a computer-based testing in which a customized software was used to present a flickering target on the screen. The methods generally followed procedures described by Dimitrov 2011. The subject sat at 1 m from the computer screen and wore refractive correction corrected for 1 m distance. Only the study eye was tested, and the non-tested eye remained occluded throughout the testing. The stimuli comprised of a series of flickering lights presented at three temporal frequencies; low (< 10 Hz), mid (10 to 20 Hz) and high (> 20 Hz) and two background luminance levels in the upper mesopic range. Each stimulus varied in contrast with the background. The stimulus with brightest contrast was initially presented which then progressively decreased. A staircase method was used to determine the subject’s threshold. The contrast at which a subject can no longer perceive the stimulus was identified as the threshold. Contrast threshold can range from 0 (best) to 1 (worst).

#### Color sensitivity

Color sensitivity tests were performed using a computer program. Thresholds were determined using red, green and blue Landolt C stimuli with varying saturation presented on an equiluminant background. Subjects were tested at three luminance levels in the low photopic/high mesopic range.

#### Computer based static contrast sensitivity test

Contrast sensitivity was measured using a computer program. With a fixed spatial frequency (0.5 and 5 cycles per degree) static stimuli, contrast between the stimulus and the background was altered until the stimulus was no longer visible. The test was performed under a regular condition and under a low luminance condition (using 1.2 ND).

#### Lateral inhibition

Subjects viewed a Hermann grid on a computer monitor. The grid consisted of an 8 × 8 square pattern of 64 black squares. The squares were presented against a white background. Subjects were asked to confirm their ability to see the illusory dark shadows at the intersection of the squares. The contrast of squares to background was then incrementally reduced until the subject reported that the illusory patches were no longer visible. The value of contrast was then recorded.

#### Shape discrimination

Three circular patterns were presented on the monitor. Two patterns were exactly circular and one was distorted by a sinusoidal variation along the angular direction. The subject was asked to identify the distorted pattern. If the subject identified the correct pattern the amplitude of the angular distortion was reduced. A staircase procedure was used to determine the threshold for each subject.

#### Pelli-Robson test

Contrast sensitivity was measured using the Pelli-Robson chart at 1 m [27].

#### Questionnaire

The questionnaire comprised of 4 questions on the extent of difficulties that a subject experiences during 1) night driving 2) oncoming head lights 3) reading in dim light and 4) adjusting to see in dim lit.

#### Test-retest repeatability and reproducibility

A subset of the study subjects (*N* = 21; *N* = 7 normals, *N =* 7 non-advanced AMD with good prior VCF threshold and *N =* 7 non-advanced AMD with reduced VCF threshold) were brought in to assess test-retest repeatability and reproducibility for the Ora-VCF™ test and Ora-tablet LL 2.0 ND reading test. Subjects brought in for this sub-study were randomly selected from the larger cohort. To assess repeatability, tests were performed twice on the same day (Day 1) with 1 h interval between the two tests. Reproducibility was assessed by repeating the same tests 2 weeks later (Day 2). All subjects were tested around the same time during Day 1 and Day 2 to minimize diurnal effects on test outcomes. All tests were administered by the same examiner during both days and none of the subjects had any change in medical or ocular conditions between the 2 days.

## Statistical analysis

Descriptive analyses and group comparisons for visual function tests were conducted using R 3.4.3. ROC analyses were conducted using the pROC R package. Test-retest repeatability and reproducibility were analyzed using SAS 9.4 PROC GLIMMIX.

Group means were compared using t-test and reported as mean ± standard error (SE) of the mean. As this was an exploratory study, no adjustment for multiple testing was made. As sensitivity tests, group means were also compared using a Wilcoxon rank-sum test and using a t-test after excluding AREDS 3 and 4 subjects from the non-advanced AMD group. In order to compare the abilities of individual tests in differentiating normal from non-advanced AMD, receiver operating characteristic (ROC) curve analysis was conducted. For each test we calculated the percent sensitivity at 80% specificity, which provides the true positive rate for the test if the threshold for a positive test result were set to yield a 20% false positive rate. For each test we also calculated two measures of overall discriminative ability: the area under the ROC curve (AUC) and the Youden index. These measures range between 0 and 1, with higher values indicating greater overall discriminative ability [28]. While these measures are informative, we caution that the AUC is not clinically meaningful because its value is not associated with a fixed positivity threshold, and the Youden index may be based on a threshold that is not clinically relevant [29]. The standard error and 95% confidence interval for the AUC was calculated using DeLong’s method [30]. Correlation analysis used Pearson’s product moment correlation coefficient and Fisher’s Z transform for statistical tests. Test-retest repeatability (repeat on the same day) and reproducibility (repeat 2 weeks later) was assessed on a subset of 21 subjects. Positivity was evaluated for each subject and assessment based upon threshold levels that were fixed at the 80% specificity level (i.e., 80% of normals score worse than that value). Percent agreement for repeatability was for tests repeated on the same day; percent agreement for reproducibility was measured for tests repeated during the same session on different days. Percent agreement was estimated using a repeated measures logistic regression model. Comparison of means in the repeatability and reproducibility subset were conducted using LS Means from a repeated measures model with random effects for subject and day within subject.

## Results

**Fig. 2:**
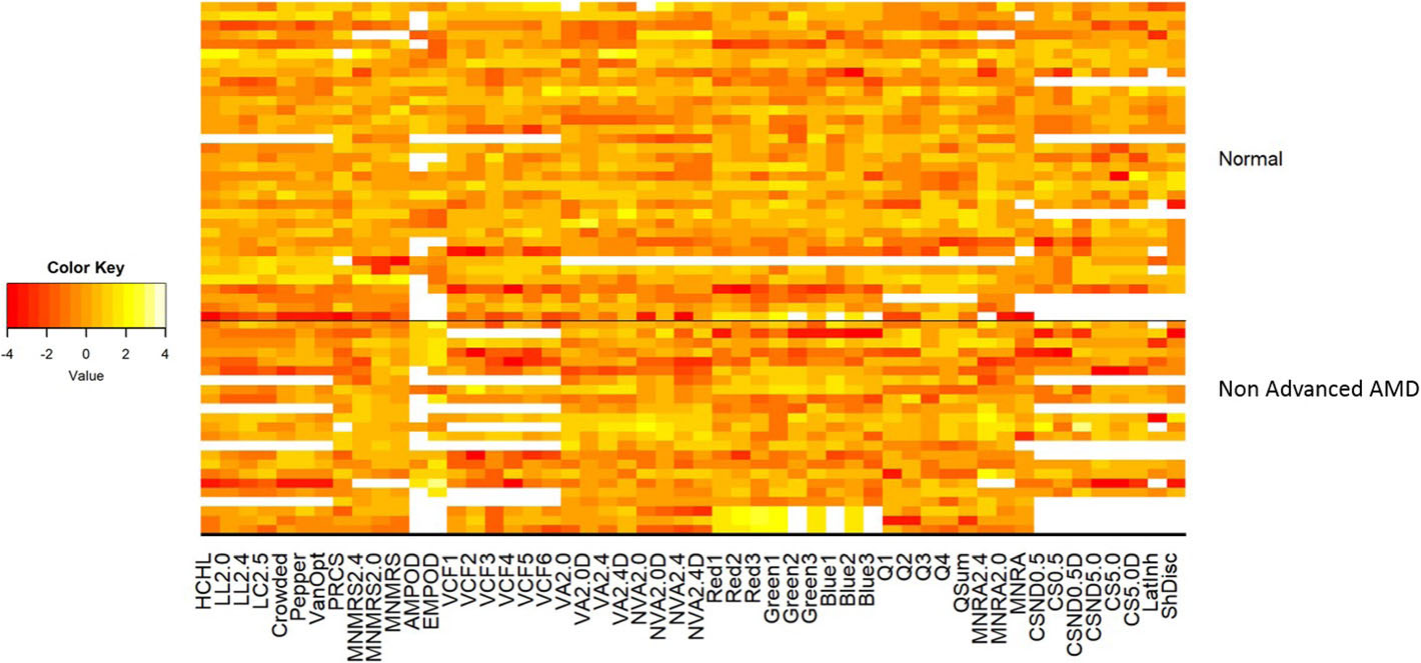
Heat map displaying all tests performed on all subjects. Color key values indicate number of SD from the mean based on normal subjects, truncated to ±4. Negative values (red) indicate scores that are worse than the normal mean; positive values (yellow) indicate scores that are better than the normal mean; white indicates missing values

Fifty seven subjects were included in the study comprising of thirty four normal controls and 23 AMD subjects. The mean age (±SD) for the normal group was 74.6 ± 4.7 years and for the AMD group was 74.3 ± 6.7 years (*p* = 0.84). Subject demographic details are summarized in Table 1. Figure 2 shows a heat map of all tests performed on all subjects. The tests evaluated in the current study were chosen after a detailed literature search and narrowing down on specific tests and parameters to identify underlying visual dysfunction in early stages of AMD.

**Table 1:** Demographics Details

	Normal *N* = 34	Non-advanced AMD *N* = 23
Age, mean ± SD, years	74.6 ± 4.7	74.3 ± 6.7
Sex	23 Females 11 Males	13 Females 10 Males
Race	32 Whites 2 Afro-Americans	23 Whites

### Current commonly used clinical endpoints: Normal vs non-advanced AMD groups

**Fig. 3:**
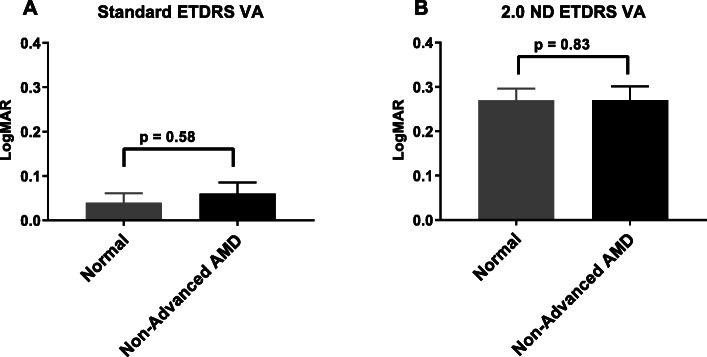
Standard ETDRS VA (**a**) and LL 2.0 ETDRS VA (**b**) in normal and non-advanced AMD groups. Error bars are standard errors

Using some current commonly used clinical endpoints we compared visual function outcomes between the study groups (Table 2). The standard ETDRS distance VA was not significantly different between the normal (mean ± SE, 0.04 ± 0.02 logMAR) and the non-advanced AMD group (mean ± SE, 0.06 ± 0.02 logMAR) (*p* = 0.57). LL ETDRS VA measured using 2.0 ND also showed no difference between the normal (mean ± SE, 0.27 ± 0.03 logMAR) and the non-advanced AMD group (mean ± SE, 0.27 ± 0.03 logMAR) (*p* = 0.84) (Fig. 3). There was no significant difference between the two groups using the MNREAD test. The mean maximum reading speed in normals was 211.93 ± 9.86 wpm compared to 216.86 ± 8.15 wpm in non-advanced AMD (*p* = 0.72). The mean reading acuity in normals was 0.11 ± 0.03 logMAR compared to 0.16 ± 0.03 logMAR in non-advanced AMD (*p* = 0.27). The Pelli-Robson log contrast sensitivity test also showed no significant difference between the normal group (1.76 ± 0.04) and the non-advanced AMD group (1.67 ± 0.05) (*p* = 0.14).

**Table 2:** Visual function outcomes using some current commonly used endpoints. Descriptive analysis and group comparison

Tests	Normal (mean ± SE)	Non-advanced AMD (mean ± SE)	*P* Value
ETDRS VA	0.04 ± 0.02	0.06 ± 0.02	0.568
ETDRS LL 2.0 ND VA	0.27 ± 0.03	0.27 ± 0.03	0.837
ETDRS LL 2.0 ND Deficit	0.22 ± 0.02	0.21 ± 0.02	0.940
MNREAD Regular Max reading speed (wpm)	211.93 ± 9.86	216.86 ± 8.15	0.723
MNREAD Regular Reading acuity	0.11 ± 0.03	0.16 ± 0.03	0.210
MNREAD LL 2.0 ND Max reading speed (wpm)	177.58 ± 12.24	203.40 ± 7.66	0.118
MNREAD LL 2.0 ND Reading acuity	0.34 ± 0.04	0.32 ± 0.04	0.782
Pelli-Robson CS	1.76 ± 0.04	1.67 ± 0.05	0.145
*P*-values calculated using a two-sided two-sample t-test

### Novel tests developed for the current study: Normal vs non-advanced AMD groups

**Fig. 4:**
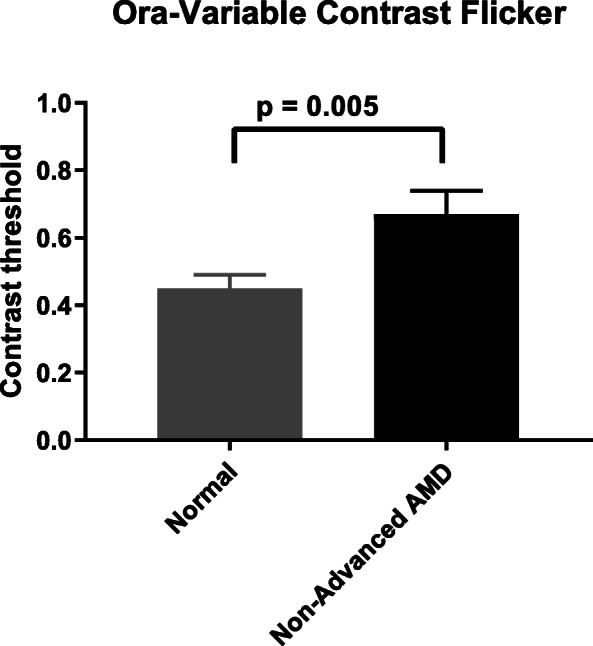
Ora-VCF™ test in normal and non-advanced AMD

**Fig. 5:**
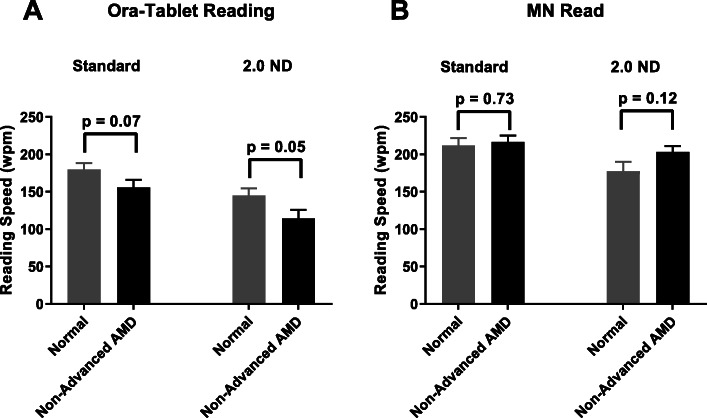
Reading speed using Ora-tablet reading test (**a**) and MNREAD test (**b**) in normal and non-advanced AMD groups. Error bars are standard errors

As described in the methods, we developed a battery of visual function tests to identify endpoints that can differentiate the non-advanced AMD group with good visual acuity from age-matched normal controls. In our cohort, two tests showed promise as potential endpoints. The Ora-VCF™ test produced the best results in differentiating the non-advanced AMD from normal group (Table 3). Using a low mesopic, high frequency flickering stimulus, AMD subjects exhibited significantly worse visual function (i.e. higher contrast threshold) than normals (0.67 ± 0.07 in non-advanced AMD vs 0.45 ± 0.04 in normal *p* = 0.005) (Fig. 4). This difference remained statistically significant using the Wilcoxon rank-sum test (*p* = 0.013) and after adjusting for standard ETDRS distance VA (*p* = 0.009). The difference also remained statistically significant when AREDS 3 and 4 subjects were excluded from the non-advanced AMD group (0.67 ± 0.07, *p* = 0.008 vs normal). The Ora-VCF™ test with high mesopic, high frequency stimulus also had a worse threshold in non-advanced AMD but did not reach significance (0.33 ± 0.04 in non-advanced AMD vs 0.26 ± 0.02 in normal *p* = 0.08). Reading challenges using Ora-tablet showed that reading performance under low luminance conditions (using 2.0 ND) was significantly worse in non-advanced AMD subjects compared to normal controls. Tablet reading speed using LL 2.0 ND was 114.55 ± 11.22 wpm in non-advanced AMD compared to 145.17 ± 9.55 wpm in normals (*p* = 0.049) (Fig. 5). This difference remained statistically significant when using the Wilcoxon rank-sum test (*p* = 0.024). When AREDS 3 and 4 subjects were excluded from the non-advanced AMD group, the group difference was similar in magnitude but was no longer statistically significant (114.45 ± 13.89 wpm, *p* = 0.077). LL 2.0 ND reading speed had moderate negative correlation with the Ora-VCF™ test with the low mesopic, high frequency flickering stimulus (*r =* − 0.48, *p* = 0.0002) and with the high mesopic, high frequency stimulus (*r =* − 0.27, *p =* 0.04). Our computer based blue color sensitivity test was worse in the non-advanced AMD group but did not reach significance (0.30 ± 0.03 in non-advanced AMD vs 0.24 ± 0.02 in normal *p* = 0.09).

**Table 3:** Visual function outcomes using new tests developed for this study. Descriptive analysis and group comparison

Tests	Normal (mean ± SE)	Non-advanced AMD (mean ± SE)	*P* Value
Ora-VCF™ test (low mesopic high freq)	0.45 ± 0.04	0.67 ± 0.07	**0.005**
Ora-VCF™ test (high mesopic high freq)	0.26 ± 0.02	0.33 ± 0.04	0.082
Ora-tablet HCHL Reading speed (wpm)	179.97 ± 8.52	155.69 ± 10.17	0.081
Ora-tablet LL 2.0 ND Reading speed (wpm)	145.17 ± 9.55	114.55 ± 11.22	**0.049**

*P*-values calculated using a two-sided two-sample t-test

**Fig. 6:**
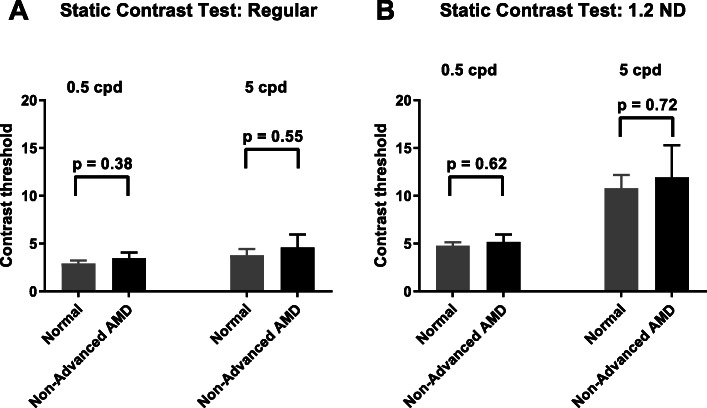
Contrast thresholds using static contrast test for 0.5 cpd and 5 cpd spatial frequencies in normal and non-advanced AMD groups. Error bars are standard errors

While some of our tests were able to differentiate non-advanced AMD from normal controls, a few of them did not show a significant difference or a trend in differentiating between the two groups. Our computer based static contrast test showed no difference between AMD and normals (Fig. 6). No difference was observed between the two groups using the shape discrimination test or the lateral inhibition test. No difference was noted in the questionnaire outcomes between the AMD and the normal group (*p* > 0.05 for all comparisons).

### ROC analysis

We conducted ROC analysis in order to compare the abilities of these individual tests in differentiating normal from non-advanced AMD (Tables 4 and 5). Percent sensitivity for standard ETDRS VA, 2.0 ETDRS VA and Pelli-Robson CS were 27.15, 17.93 and 36.78, respectively. Percent sensitivity for low mesopic, high frequency VCF and LL 2.0 tablet reading were 58.50 and 47.37 respectively. The AUC values for ETDRS VA, 2.0 ETDRS VA and Pelli-Robson CS were 0.56, 0.51 and 0.63, respectively; the AUC values for low mesopic, high frequency VCF and LL 2.0 tablet reading were 0.71 and 0.69, respectively.

**Table 4:** Visual function outcomes using some current commonly used endpoints: ROC analysis

Tests	AUC (SE)	AUC 95% CI	Youden Index	Sensitivity (%) at 80% Specificity
ETDRS VA	0.56 (0.08)	0.41–0.71	0.11	27.15
ETDRS LL 2.0 ND VA	0.51 (0.08)	0.35–0.66	0.08	17.93
ETDRS LL 2.0 ND Deficit	0.46 (0.08)	0.31–0.61	0.10	8.12
MNREAD Regular Max reading speed (wpm)	0.47 (0.08)	0.31–0.63	0.14	22.73
MNREAD Regular Reading acuity	0.62 (0.08)	0.47–0.78	0.19	34.91
MNREAD LL 2.0 ND Max reading speed (wpm)	0.36 (0.08)	0.21–0.51	0.12	9.09
MNREAD LL 2.0 ND Reading acuity	0.48 (0.08)	0.33–0.64	0.01	16.82
Pelli-Robson CS	0.63 (0.07)	0.48–0.77	0.20	36.78

**Table 5:** Visual function outcomes using new tests developed for this study: ROC analysis

Tests	AUC (SE)	AUC 95% CI	Youden Index	Sensitivity (%) at 80% Specificity
Ora-VCF™ test (low mesopic high freq)	0.71 (0.09)	0.55–0.88	0.42	58.50
Ora-VCF™ test (high mesopic high freq)	0.63 (0.08)	0.47–0.80	0.23	39.06
Ora-tablet HCHL Reading speed (wpm)	0.67 (0.08)	0.52–0.82	0.31	50.53
Ora-tablet LL 2.0 ND Reading speed (wpm)	0.69 (0.08)	0.54–0.84	0.41	47.37

### Test-retest repeatability and reproducibility

Test-retest repeatability (repeat on the same day) and reproducibility (repeat 2 weeks later) was assessed on a subset of normal and non-advanced AMD subjects. High percent agreement between the tests was seen for both high frequency, mesopic Ora-VCF™ test and Ora-tablet LL 2.0 reading test. Repeatability was 82.1% for the Ora-VCF™ test and 91.9% for Ora-tablet LL 2.0 reading test. Reproducibility was 88.9% for the Ora-VCF™ test and 89.9% for the Ora-tablet LL 2.0 reading test.

While the objective of the test-retest analyses was to examine repeatability and reproducibility, the Ora-VCF™ test with low mesopic, high frequency stimulus demonstrated worse thresholds in non-advanced AMD with reduced VCF (0.94 ± 0.10) than in non-advanced AMD subjects with good VCF (0.57 ± 0.08, *p* = 0.008) and in normal subjects (0.58 ± 0.08, *p* = 0.009). Similarly, for the Ora-tablet LL 2.0 ND reading test, non-advanced AMD subjects with reduced VCF had slower reading speeds (40.00 ± 12.77 wpm) than in non-advanced AMD subjects with good VCF (106.76 ± 11.81 wpm, *p* = 0.0005) and in normal subjects (117.42 ± 11.81 wpm, *p* < 0.0001). For both tests, there were no statistical differences between non-advanced AMD subjects with good VCF and normal subjects (*p* = 0.95 for VCF, *p* = 0.53 for Ora-tablet LL 2.0 ND reading).

## Discussion

In the current study we evaluated a group of non-advanced AMD subjects with good visual acuity and age-matched normal controls using a battery of visual function endpoints. We found that the Ora-VCF™ test and Ora-tablet 2.0 ND reading tests were able to identify significant visual dysfunction in the non-advanced AMD group while some current commonly used endpoints such as ETDRS VA, LL 2.0 ETDRS VA and MNREAD test found no significant difference between the two groups. While the cross-sectional nature of the current study allows to identify visual deficits at given time, a longitudinal study is currently in progress to assess predictive value of these tests in identifying AMD progression.

Typically AMD severity and its impact on a patient is assessed by fundus evaluation. Dilated ophthalmoscopy and fundus photo examination have now been augmented with more sophisticated imaging techniques such as high resolution OCT, fundus autofluorescence etc. [31, 32] While these imaging techniques provide excellent visualization of the extent of retinal abnormalities, they often also indicate irreversible neuronal damage. Sensitive tests of visual function can identify neuronal dysfunction before neuronal atrophy occurs and thereby can provide a window of opportunity for therapeutic intervention. Histological evidence suggests that rod photoreceptors might be affected initially followed by cone photoreceptors during non-advanced AMD [33]. Consequently a number of studies have focused on using rod-mediated dark adaptation as a potential endpoint in non-advanced AMD [34–36]. Pure rod-based functional tests have some limitations including long test duration and greater difficulty in performing the test [21].

Pure scotopic rod vision (equivalent to seeing in dark on a moonless night) is extremely rare in real life. When subjects report difficulty in night vision or in low light levels they commonly refer to mesopic function such as night driving or entering a movie theatre. In addition to the luminance of a target, contrast sensitivity is an important visual function task that is critical to distinguishing an object from its background, especially in aging and AMD. For the current study we developed a computer based contrast test under mesopic condition which would enable functional contributions from both cone and rod photoreceptors. In the Ora-VCF™ test, by using a combination of mesopic background luminance and high frequency flickering stimuli we were able to develop a contrast test that could elicit underlying dysfunction in non-advanced AMD. It is known that flickering stimuli can increase retinal neuronal metabolic demand due to underlying neurovascular coupling [37]. As AMD pathology spans across multiple retinal structures both vascular (choriocapilaries) and neuronal (photoreceptors) as well as intermediary layers (RPE and Bruch’s membrane), it is important to develop functional tests that can challenge these complex structures and elicit underlying dysfunction. Previous studies have shown that flicker sensitivity could differentiate normal and AMD subjects [21, 22, 38]. However these studies included AMD subjects with definite VA loss unlike our non-advanced AMD cohort with near normal VA. Here we looked into a range of flicker frequency and found high frequency stimuli to be the best and low frequency to be least useful in identifying dysfunction in non-advanced AMD. In order to further enhance separation between our non-advanced AMD group and age-matched normals, our flickering stimuli was presented on a mesopic luminance background. It is well documented that AMD symptoms are exacerbated in low lighting conditions [26, 39]. Interestingly LL 2.0 ND VA test was unable to differentiate normals from non-advanced AMD cohort. Similar results have been reported in other studies where low luminance VA showed no dysfunction in non-advanced AMD [18, 40] but was sensitive in more advanced forms with GA [26].

Reading tests have been widely used in AMD studies but the majority of the studies have been done in advanced AMD focusing on methods to improve reading performance in subjects with central scotoma [41, 42]. Reading speed as an outcome to assess reading performance in non-advanced AMD has shown some promising results recently [17, 43]. Even in the absence of absolute scotoma, reading continuous texts accurately is more challenging than identifying individual letters from a VA chart. This could be further challenged by altering the testing conditions such as luminance, contrast, materials etc. In the current study, reading performance was assessed using an electronic tablet device. Ora-tablet reading test using 2.0 ND identified significant visual dysfunction in the non-advanced AMD group compared to normals. In contrast, no difference between the two groups was observed using the standard MNREAD test or LL 2.0 MNREAD test. While we would like to interpret this result with caution, there are a few important distinctions between our reading test and the MNREAD test. First, the MNREAD test was tested with a standard paper based chart while Ora-tablet reading test was administered using an electronic tablet device and this could have contributed to better luminance control. Second, the MNREAD passages were smaller (about 10 words per passage) whereas our passages were a bit longer. Reading speed using smaller passages could result in larger variability than with longer passages.

As the Ora-VCF™ test and Ora-tablet reading tests differentiated between non-advanced AMD and normal subjects, it is natural to consider the development of a multi-factor endpoint to improve differentiation. Uncorrelated tests offer the best potential to yield significant improvements. With our tests, the moderate correlation between the Ora-VCF™ test and Ora-tablet reading tests is a limiting factor. Although we have observed that some modest improvements in differentiation are possible by combining our endpoints, this remains an area of continued research.

While the Ora-VCF™ test and Ora-tablet reading tests were able to identify significant differences between the non-advanced AMD and age-matched normal group, a few of our newly developed tests did not. Looking at tests that failed are equally important in understanding factors that contribute towards development of good tests. In the static contrast sensitivity test, we used 2 different spatial frequencies 0.5 and 5 cycles per degree. These correspond to VA of about 20/1200 to 20/120 which were probably too large given the relatively unaffected VA in our non-advanced AMD cohort. Also the low luminance setting was in the photopic range (about 6 cd/m^2^) which was probably not challenging enough to differentiate the two groups. Similarly our shape discrimination targets were also fairly large (about 3 degrees) and was unable to differentiate between the two groups. For the lateral inhibition test, we used the Hermann grid presented on the monitor. However a large number of subjects, both from the normal and non-advanced AMD groups, were unable to perceive the grey circular illusion even with repeated instructions and demos. This explains the lack of differences seen between the groups using this test. Our brief questionnaire focused on comparing the extent of difficulties experienced by subjects when performing some common tasks such as driving and reading under mesopic conditions. However we found that our cohort of non-advanced AMD subjects were experiencing similar symptoms as normal controls. Most of the subjects, both normals and AMD, reported experiencing mild to moderate difficulty in performing such tasks and hence no significant difference between the two groups was noted. It is important to note that a possible drawback is that we used a very brief questionnaire comprising of 4 questions only and the results could have been different had we used a more elaborate questionnaire such as NEI-VFQ.

Our results are in general agreement with similar studies in the literature, although differences in study methods, subject population, study inclusion criteria as well as specific visual function tests used in different studies makes a direct comparison challenging. Dimitrov et al. assessed a battery of visual function tests and concluded that contrast thresholds assessed using flickering targets could be used as optimal tests to assess visual function in AMD [21]. In a recently published study by Pondorfer et al. [23], several visual function tests were found to be sensitive in differentiating normal from intermediate AMD subjects. While their general conclusion that contrast sensitivity and mesopic testing conditions are sensitive to elicit visual dysfunction in non-advanced AMD, are in general agreement with the results of our study, there are several differences in study methods between our study and Pondorfer et al’s. First, the subject. Population used for both AMD and normal group in Pondorfer et al. are much younger than used in our study (mean age 75 years in our study for both normal and AMD vs 62 years for normal and 69 years for AMD group in Pondorfer et al). Second, normals in the Pondorfer study had better VA than our normal cohort and AMD in their study had worse VA than our cohort. Visual function tasks such as reading and contrast sensitivity are known to be impacted due to normal aging as well as visual acuity abilities. Finally, in Pondorfer et al’s study the entire AMD group comprised of intermediate AMD. However, in our study when subjects were grouped in the similar methods as used in their study, about half of our AMDs are early AMD and the other half are intermediate AMD. These differences in study design could explain more robust results seen by Pondorfer et al. in a variety of visual function tests used.

It is important to note that this not a validation study but is an exploratory study to identify the most promising test or tests that could be potentially used for AMD clinical trials. It is critical that after identifying the most promising test, multiple, independent studies must to be carried out with rigorous study design and statistical methods in order to evaluate if a test has the sensitivity as well as clinical meaningfulness to be used a pivotal endpoint in clinical trials or a clinical test in eye care setting.

In summary, given that our cohort of AMD subjects were much earlier in disease progression with well-preserved visual acuity compared to most endpoint studies done in AMD, it is not surprising that only a few of our tests were able to differentiate the non-advanced AMD group from age-matched normals while a few of them could not. An ideal endpoint such as Ora-VCF™ test should subtly challenge the retina. Normals should be able to perform well even under this subtle challenge while non-advanced AMDs with underlying neuronal dysfunction will exhibit visual deficits. Non sensitive tests such as our static contrast sensitivity test had a challenge that was too easy for both normals and non-advanced AMD groups and the lateral inhibition challenge was too hard for both the groups.

## Conclusion

In the current study we evaluated a battery of visual function tests to identify sensitive outcomes in non-advanced dry AMD subjects with good visual acuity. We found that the Ora-VCF™ test and Ora-tablet LL 2.0 ND reading test were able to significantly differentiate non-advanced AMD cohorts from age-matched normal controls. No significant difference between the two groups was found using some current commonly used endpoints such as ETDRS VA, 2.0 ETDRS VA and MNREAD test. Ora-VCF™ test and Ora-tablet LL 2.0 ND reading tests show promise as potential endpoints for therapeutic intervention in non-advanced AMD trials. Robust psychophysical functional end points could provide opportunity for reversibility with appropriate therapeutic intervention in non-advanced AMD. They could also additionally aid in high throughput subject screening and result in shorter study duration. This is especially important in a multifactorial disease like AMD in which the exact pathological source remains uncertain.

## Data Availability

The datasets used and/or analyzed during the current study are available from the corresponding author on reasonable request.

## Abbreviations

AMD: Age-related macular degeneration
AUC: Area under the curve
ETDRS: Early treatment diabetic retinopathy study
GA: Geographic atrophy
HCHL: High contrast high luminance
LL: Low luminance
ND: Neutral density
OCT: Optical coherence tomography
ROC: Receiver operating characteristic
RPD: Reticular pseudo drusen
RPE: Retinal pigment epithelium
VA: Visual acuity
VCF: Variable contrast flicker
wpm: Words per minute
